# Gomisin G Inhibits Pancreatic Cancer Cell Growth by Promoting YAP Degradation

**DOI:** 10.1002/fsn3.72111

**Published:** 2026-07-25

**Authors:** Lan Li, Jiayu Chen, Jiayi Shao, Lingxiao Ye, Shuya He, Ju Huang, Licai He, Jiawei Cao, Haihua Gu, Guang Wu

**Affiliations:** ^1^ School of Public Health Wenzhou Medical University Wenzhou China; ^2^ Key Laboratory of Laboratory Medicine, Ministry of Education, School of Laboratory Medicine and Life Sciences Wenzhou Medical University Wenzhou China; ^3^ Wenzhou Key Laboratory of Cancer Pathogenesis and Translation, Key Laboratory of Laboratory Medicine, Ministry of Education, School of Laboratory Medicine and Life Sciences Wenzhou Medical University Wenzhou China

**Keywords:** apoptosis, cell cycle, Gomisin G, LATS1, pancreatic cancer, YAP

## Abstract

Pancreatic cancer is highly lethal, with a five‐year survival rate of less than 5%. Gomisin G, extracted from the fruit of *Schisandra chinensis*, is known for its anti‐tumor, anti‐inflammatory, and antioxidant properties. However, its specific effects on pancreatic cancer and the underlying mechanisms remain unclear. In this study, we used a colony formation assay to evaluate the impact of Gomisin G on pancreatic cancer cell proliferation and colony formation. Flow cytometry and western blot analyses revealed that Gomisin G induces G1 phase arrest by modulating the expression of cyclin D1, p21, p27, and Rb activity, and triggers apoptosis in pancreatic cancer cells. Using a mouse xenograft model, we found that Gomisin G significantly inhibits the growth of PANC‐1 pancreatic tumors. Mechanistically, RNA‐seq, western blot, qPCR, and confocal microscopy showed that Gomisin G promotes LATS1‐mediated YAP phosphorylation, accelerates YAP protein degradation, reduces its nuclear localization, and down‐regulates the mRNA levels of the target genes CTGF and CYR61 in the Hippo‐YAP pathway. Overall, our findings suggest that Gomisin G has significant anti‐cancer activity against pancreatic cancer and may be a promising candidate for therapeutic development.

## Graphical abstract

This study evaluated the inhibitory effect of Gomisin G, a natural dibenzocyclooctadiene lignan isolated from the fruit of Schisandra chinensis (Turcz.) Baill., on pancreatic cancer cell growth. We found that Gomisin G activated LATS1 kinase and promoted YAP phosphorylation. Phosphorylated YAP was retained in the cytoplasm and degraded, preventing its nuclear translocation and suppressing its transcriptional co‐activator activity. This reduced the expression of downstream targets CTGF and CYR61, leading to cell cycle arrest and apoptosis, ultimately inhibiting tumor growth. The figure was created with BioGDP.com.

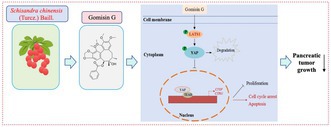

## 1 Introduction

Pancreatic cancer remains one of the leading causes of cancer‐related deaths globally. Despite advances in surgery and chemotherapy, the overall prognosis has not improved significantly over the past decades. The median survival for patients with pancreatic cancer is approximately 6 months, and the five‐year survival rate is less than 5% (Carrato et al. 2015). Factors such as obesity, high‐fat diets, diabetes, smoking, alcohol dependence, and chronic pancreatitis contribute to the rising incidence of pancreatic cancer each year (Siegel et al. 2016). The absence of early symptoms and reliable diagnostic markers leads to most patients being diagnosed at an advanced stage. Less than 20% of these patients have potentially resectable diseases. Moreover, even with curative resection, the majority will experience relapse and ultimately succumb to the disease. Standard chemotherapy for advanced pancreatic cancer extends survival by only approximately 3 months (Faris et al. 2013; Von Hoff et al. 2013). The complex interactions between the cancer cells and the surrounding stroma further complicate treatment efforts (Ansari et al. 2017). Therefore, exploring new therapeutic approaches is critically needed.

*Schisandra chinensis*, belonging to the Magnoliaceae family, is renowned in traditional Chinese medicine (Szopa et al. 2017; Yang et al. 2022). Its primary constituents include volatile components, lignans, organic acids, polysaccharides, and glycosides (Yang et al. 2022). The Schisandrin fruit notably contains a variety of lignans such as Schisandrin A, B, C, Schisandrol A, B, Schisantherin A, B, C, D, and Gomisin variants D, E, G, H, J, M, N, O, S, U, among others (Ikeya et al. 1979). Gomisin G, a dibenzocyclooctadiene lignan naturally occurring in *Schisandra chinensis* fruit, has garnered increasing attention as a bioactive food constituent with diverse health‐promoting properties, encompassing hepatoprotective, anti‐inflammatory, and anti‐tumor effects (Chen et al. 1997; Ehambarampillai and Wan 2025; Ryu et al. 2011; Xiaoyang et al. 2015). Mechanistic investigations indicate that the antineoplastic efficacy of this compound is mediated principally through suppression of the PI3K/AKT signaling axis. In colorectal cancer Lovo cells, Gomisin G treatment markedly attenuates AKT phosphorylation, thereby blocking downstream oncogenic signaling and triggering both apoptotic cell death and cell cycle arrest, concomitant with diminished expression of cyclin D1 and Rb protein (Maharjan et al. 2019). Comparable anti‐proliferative outcomes have been documented in TNBC, wherein this lignan similarly reduces AKT phosphorylation and suppresses cyclin D1 and Rb levels (Maharjan et al. 2018). Dysregulation of the PI3K/AKT pathway represents a hallmark of pancreatic carcinogenesis; oncogenic KRAS mutations and PTEN loss frequently cooperate to drive constitutive pathway activation, fostering aggressive tumor growth, metastatic dissemination, and therapeutic resistance (Stanciu et al. 2022). Notably, the anticancer potential of *Schisandra* lignans extends beyond Gomisin G‐related congeners, including Gomisin A, N, and J, which have demonstrated broad‐spectrum anticancer efficacy against multiple solid malignancies (Hwang et al. 2011; Jung et al. 2019; Yim et al. 2009). While the anti‐tumor mechanisms of Gomisin G are well characterized in colorectal and breast cancer models, its specific biological impact on pancreatic cancer cells and the associated molecular underpinnings remain largely unexplored.

Pancreatic cancer is a complex disease characterized by various genomic, proteomic, and epigenetic changes, which influence several key signaling pathways (Torres and Grippo 2018). The Hippo signaling pathway plays an important role in tumor development. Initially identified in fruit flies, this pathway is highly conserved in mammals. Its core components in mammals include the kinase cascade of MST1/2 (homologous to Drosophila Hippo) and LATS1/2. The MST1/2 complex, together with the scaffold protein WW45, phosphorylates and activates LATS1/2 kinase. The activated LATS1/2 then associates with the regulatory protein MOB, which phosphorylates and inhibits YAP and TAZ, the primary downstream effectors of the Hippo pathway. These effectors regulate genes critical for cell proliferation and survival. Phosphorylation by LATS1/2 retains YAP/TAZ in the cytoplasm and leads to their ubiquitin‐mediated degradation, thereby inhibiting their activity (Moroishi et al. 2015; Wang et al. 2020; Yu et al. 2015).

In this study, we assessed the efficacy of Gomisin G in pancreatic cancer cell lines, demonstrating that Gomisin G inhibited their growth. We also explored its mechanism of action in these cells. Gomisin G induced cell cycle arrest at the G1 phase and promoted apoptosis through a cyclin D1‐dependent mechanism. Additionally, it reduced mRNA levels of the downstream target genes CTGF and CYR61 in the Hippo signaling pathway by modulating the LATS1‐YAP axis. Our findings suggest that Gomisin G holds promise as a natural‐product candidate for treatment of pancreatic cancer.

## 2 Materials and Methods

### 2.1 Gomisin G

Gomisin G is a naturally occurring dibenzocyclooctadiene lignan isolated from the fruit of *Schisandra chinensis* (Turcz.) Baill. The compound possesses a chiral dibenzocyclooctadiene skeleton and is systematically named as (6S,7S,8S)‐7‐hydroxy‐1,2,3,13‐tetramethoxy‐6,7‐dimethyl‐5,6,7,8‐tetrahydrobenzo[3′,4′]cycloocta[1′,2′:4,5]benzo[1,2‐d][1,3]dioxol‐8‐yl benzoate (CAS 62956–48‐3, molecular formula C_30_H_32_O_9_). It was purchased from MedChemExpress Ltd. (Cat#HY‐N0858, Monmouth Junction, NJ, USA). Its purity was determined using an Agilent 1260 series high performance liquid chromatography (HPLC) system equipped with a C18 column (3.5 μm, 4.6 × 50 mm) at room temperature. A UV–Vis detector (Agilent Technologies, Santa Clara, CA, USA) was employed with detection at 254 nm. The mobile phase consisted of solvent A (water containing 0.01% TFA) and solvent B (acetonitrile containing 0.01% TFA), and the flow rate was 1.8 mL/min. The purity was determined to be 99.90% based on peak area normalization. According to the product information provided by MCE, Gomisin G is soluble in dimethyl sulfoxide (DMSO) at concentrations ≥ 50 mg/mL (~93.18 mM). For in vitro experiments, Gomisin G was dissolved in DMSO to prepare a 10 mM stock solution for use in subsequent cell‐based assays. The DMSO concentration in the control group was maintained at ≤ 0.1%.

### 2.2 Antibodies

The following antibodies were sourced from Cell Signaling Technology (Danvers, MA, USA): anti‐LATS1 (Catalog No. 3477S, 1:1000), anti‐phospho‐LATS1 (Catalog No. 8654S, 1:1000), anti‐phospho‐YAP (Catalog No. 13619S, 1:1000), anti‐YAP (Catalog No. 14074S, 1:1000), anti‐CDK4 (Catalog No. 12790S, 1:1000), anti‐CDK6 (Catalog No. 13331S, 1:1000), anti‐cyclin D1 (Catalog No. 55506S, 1:1000), anti‐p21 (Catalog No. 2947S, 1:1000), anti‐p27 (Catalog No. 3686S, 1:1000), anti‐Rb (Catalog No. 9313S, 1:1000), anti‐phospho‐Rb (Catalog No. 9308S, 1:1000), anti‐β‐actin (Catalog No. 3700S, 1:5000), anti‐cleaved PARP (Catalog No. 5625S, 1:1000), anti‐PARP (Catalog No. 9542S, 1:1000), anti‐cleaved caspase‐9 (Catalog No. 7237P, 1:1000). The anti‐N‐p62 antibody (Catalog No. 610497, 1:1000) was obtained from BD Biosciences (Franklin lakes, NJ, USA).

### 2.3 Cell Culture

Human pancreatic cancer cell lines BxPC3, SW1990, PANC‐1, and CFPAC‐1 were procured from American Type Culture Collection (ATCC, Manassas, VA, USA). BxPC3, SW1990, and PANC‐1 cells were cultured in Dulbecco's modified Eagle's medium (DMEM, Thermo Fisher Scientific), while CFPAC‐1 cells were maintained in Iscove's modified Dulbecco's medium (IMDM, Thermo Fisher Scientific). All culture media were supplemented with 10% fetal bovine serum (Thermo Fisher Scientific), penicillin (100 U/mL), and streptomycin (100 μg/mL). Cells were incubated at 37°C in a 5% CO_2_ atmosphere.

### 2.4 Western Blotting Analysis

For immunoblotting, cell lysates were boiled, separated by SDS‐PAGE, and transferred onto PVDF membranes (Millipore, Billerica, MA, USA). The membranes were blocked with 5% non‐fat skim milk in TBS‐T for 1 h at room temperature. They were then probed with primary antibodies and HRP‐conjugated secondary antibodies (Jackson ImmunoResearch Laboratories, West Grove, PA, USA) and developed using chemiluminescence (ECL) reagent. Immunoreactive proteins were detected using the ChemiDoc MP imaging system (Bio‐Rad) and analyzed with Image Lab 5.0 software (Bio‐Rad).

### 2.5 Cell Proliferation and Colony Formation Assay

To assess the impact of Gomisin G on pancreatic cancer cell proliferation, cells were seeded in 48‐well plates (3 × 10^3^ cells/well) and treated with varying concentrations of Gomisin G for 3 days. For colony‐forming ability, cells were seeded in a 6‐well tissue culture plate (300 cells/well) and treated with concentrations of Gomisin G for 10 days. Cell and colonies were fixed with 4% paraformaldehyde and stained with 0.5% crystal violet solution at room temperature for 20 min. The stained crystal violet was solubilized with 10% acetic acid, and absorbance was measured at 540 nm using a microplate reader (Molecular Devices, San Jose, CA). The inhibitory concentration (IC_50_) for Gomisin G was determined using SPSS statistics software.

### 2.6 Cell Cycle Analysis

Pancreatic cancer cells were treated with Gomisin G for 24 h, harvested by trypsinization, washed with cold PBS, and fixed with 70% ethanol at 4°C for 2 h. The fixed cells were washed with PBS and incubated with Propidium iodide (Dojindo, Kumamoto, Japan) at 37°C in the dark for 30 min. Cell cycle distribution was analyzed by flow cytometry (NovoCyte, ACEA Biosciences, Hangzhou, China) using NovoExpress software.

### 2.7 Apoptosis Assay

Cells were seeded in 6‐well plates and treated with various concentrations of Gomisin G. After 72 h, cells were harvested with trypsin–EDTA, washed with FACS buffer (PBS containing 1% FBS), and stained with annexin V and propidium iodide (Dojindo) for 15 min at room temperature in the dark. Apoptosis cells were analyzed by Flow cytometry (NovoCyte, ACEA Biosciences) using NovoExpress software.

### 2.8 RNA‐Seq and Data Analysis

SW1990 cells were treated with 10 μM Gomisin G or vehicle for 24 h in biological duplicates. Total RNAs was extracted and sequenced by Novogene (Beijing, China). RNA quality was assessed using the RNA Nano 6000 Assay Kit on the Bioanalyzer 2100 system (Agilent Technologies, CA, USA). Differential expression analysis was performed using DESeq2 R software (version 1.20.0), with *p*‐values adjusted using the Benjamini and Hochberg's method to control false discovery rate. Genes with an adjusted *p*‐value < 0.05 were classified as differentially expressed.

### 2.9 Real‐Time PCR

Total RNA was extracted from cell lines using Trizol reagent (Invitrogen, Camarillo, CA, USA). Quantitative real‐time PCR was performed to measure the mRNA levels of CTGF and CYR61. cDNA was synthesized from 1 μg of purified RNA using Reverse Transcription Kit (Qiagen, Hilden, Germany) and subjected to qRT‐PCR for analysis using SYBR Green master mix (Vazyme, Jiangsu, China). The primers used were: CTGF forward 5′‐ACCGACTGGAAGACACGTTTG‐3′, reverse 5′‐CCAGGTCAGCTTCGCAAGG‐3′; CYR61 forward 5′‐AGCCTCGCATCCTATACAACC‐3′, reverse 5′‐TTCTTTCACAAGGCGGCACTC‐3′; GAPDH forward 5′‐GCAAATTCCATGGCACCGT‐3′, reverse 5′‐TCGCCCCACTTGATTTTGG‐3′.

### 2.10 Confocal Microscopy

Pancreatic cancer cells were seeded on Poly‐L‐lysine‐coated glass cover‐slips in 24‐well plates. Cells were blocked with 3% BSA and incubated with anti‐YAP antibody (Catalog No. 14074, 1:300, Cell Signaling) at room temperature for 2 h. After washing with PBS containing 1% BSA, cells were incubated with Alexa Flour 488‐conjugated goat anti‐rabbit IgG antibody (Jackson ImmunoResearch) for 1 h. Samples were mounted and observed under the Nikon A1 confocal microscope system (Nikon, Tokyo, Japan).

### 2.11 Xenograft Model

Five‐week‐old female Balb/c nu/nu mice were obtained from Charles River Laboratories (Beijing, China) and maintained under specific pathogen‐free conditions. This study strictly adhered to the principles of laboratory animal welfare and ethics. All animal experimental protocols were approved by the Institutional Animal Care and Use Committee of Wenzhou Medical University (Permit Number: wydw2024‐0182). Animals were housed in an SPF (Specific Pathogen‐Free) barrier facility at the Laboratory Animal Center of Wenzhou Medical University under the following conditions: temperature 20°C–23°C, relative humidity 50%–60%, a 12 h/12 h light/dark cycle, and five animals per cage. Animal health status was continuously monitored throughout the experiment. Clear humane endpoint criteria were established, including: body weight loss 20%, tumor ulceration or necrosis, marked impairment of mobility or feeding ability, and maximum tumor diameter exceeding 2.0 cm (approximately 1500–2000 mm^3^) or tumor volume exceeding 10% of the animal's body weight. The experiment was immediately terminated when any of the above criteria were met. Euthanasia was performed by carbon dioxide (CO_2_) overdose inhalation.

The in vivo dose of Gomisin G was selected based on prior efficacy data (1 mg/kg/day in muscle atrophy models (Yeon et al. 2022)), toxicological data for structurally related Gomisin D (50 mg/kg/day, no toxicity; Wang et al. 2023), and in vitro anti‐tumor activity (IC_50_ < 10 μM for proliferation; 3 μM for colony formation). PANC‐1 cells (5 × 10^6^) were resuspended in 50% Matrigel (BD Biosciences) and injected subcutaneously into the dorsal right flank of female BALB/c nu/nu mice. When the tumor volume reached approximately 180 mm^3^, mice were stratified by tumor size, paired, and randomly divided into two groups (5 mice per group) with comparable initial mean tumor volumes: vehicle control and Gomisin G (3 mg/kg). Mice were treated by oral gavage every three days; the treatment group was given Gomisin G formulation, while the control group received an equivalent volume of DMSO‐containing vehicle. Gomisin G formulation was prepared following the MCE protocol: a 2.5 mg/mL stock solution was made by dissolving Gomisin G in DMSO (10%) and corn oil (90%). For each mouse, the required dose was calculated based on body weight at a dose of 3 mg/kg, and the corresponding stock volume was diluted with corn oil to a final volume of 200 μL for oral administration. The vehicle control contained the same DMSO concentration (≈1.2%). Tumor diameters were monitored every 7 days using calipers throughout the experimental period. Tumor volumes were calculated using the formula: width^2^ × length/2. Mice were sacrificed 35 days after initiating Gomisin G treatment, and the tumors were excised and weighed.

### 2.12 Statistical Analysis

Data are presented as mean ± standard deviation. Statistical comparisons between two groups were conducted using the unpaired two‐tailed Student's *t*‐test. For comparisons among multiple groups, one‐way ANOVA with Bonferroni's multiple comparison test was applied. All statistical analyses were performed using Prism software (Graph Pad Software Inc). A *p*‐value less than 0.05 was considered statistically significant. Each experiment was repeated at least three times to ensure reproducibility.

## 3 Results

### 3.1 Gomisin G Inhibits the Growth of Pancreatic Cancer Cells

**FIGURE 1:**
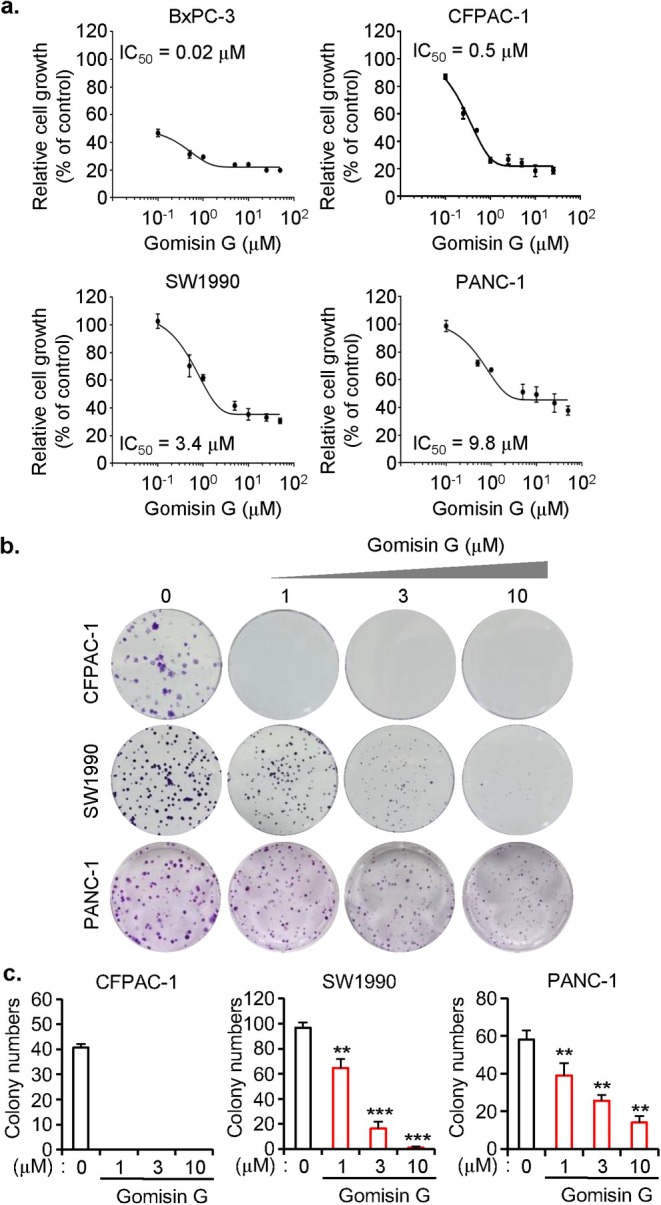
Gomisin G inhibits the growth of pancreatic cancer cells. (a) BxPC‐3, CFPAC‐1, SW1990, and PANC‐1 cells were exposed to various concentrations of Gomisin G for 3 days. Cell growth was evaluated using crystal violet staining, with absorbance measured at 540 nm to quantify relative cell growth. Non‐Gomisin G treatment cells were set as the 100% control, IC_50_ values were determined. These results are representative of three independent experiments. (b) Colony‐forming ability was assessed in CFPAC‐1, SW1990, and PANC‐1 cells treated with Gomisin G or DMSO for 10 days. Colonies were stained with crystal violet solution. (c) Quantification of the colony‐forming data from (b). Mean values from three experiments are presented. The number of colonies in the Gomisin G group was compared with the DMSO control group. Statistical analyses were performed using one‐way ANOVA with Bonferroni's multiple comparison test, **p < 0.01, ***p < 0.001.

To determine whether Gomisin G can inhibit the growth of pancreatic cancer cells, we conducted cellular proliferation and colony formation assays. Pancreatic cancer cell lines (BxPC‐3, CFPAC‐1, SW1990 and PANC‐1) were treated with Gomisin G at concentrations of 0.1, 0.5, 1, 5, 10, 25, and 50 μM, or with a vehicle control (DMSO) for 72 h. The proliferation of BxPC‐3 cells was most significantly reduced (IC_50_ = 0.02 μM), followed by CFPAC‐1 (IC_50_ = 0.5 μM), SW1990 (IC_50_ = 3.4 μM), and PANC1 (IC_50_ = 9.8 μM) (Figure 1a). To further evaluate its anti‐proliferative activity, we performed a colony formation assay by seeding 300 cells in a 6‐well culture plate and treating them with Gomisin G. As shown in Figure 1b,c, Gomisin G significantly reduced the colony‐forming ability of pancreatic cancer cells in a dose‐dependent manner.

### 3.2 Gomisin G Induces G1 Cell Cycle Arrest in Pancreatic Cancer Cells

**FIGURE 2:**
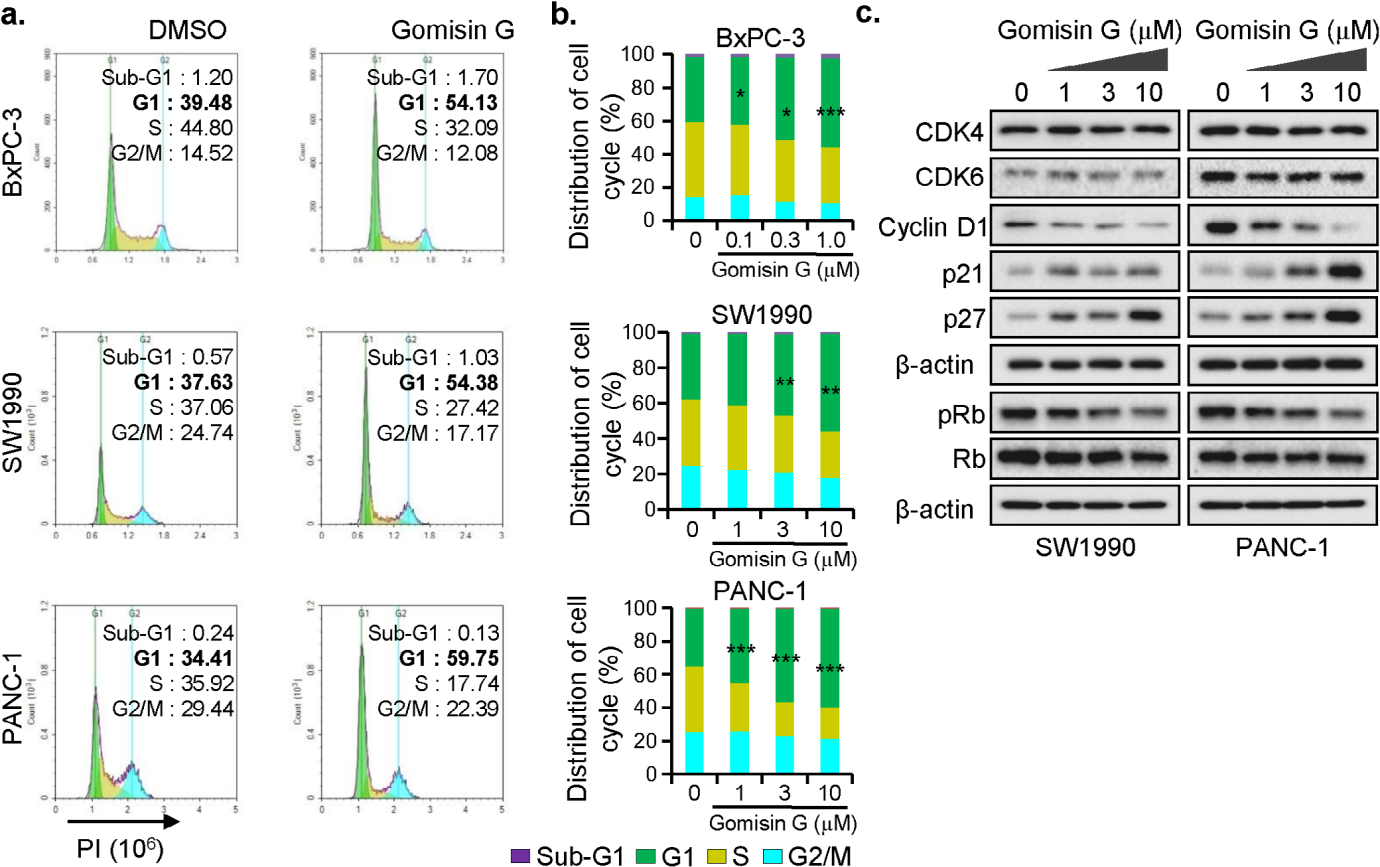
Gomisin G induces G1 phase arrest in pancreatic cancer cells. (a) Specified pancreatic cell lines were treated with various concentrations of Gomisin G for 24 h, fixed, and stained with propidium iodide. The cell cycle phases (G1, S, and G2/M) were analyzed by flow cytometry using NovoExpress software. (b) Quantification of flow cytometry data from (a). Mean values from four experiments are presented. The G1 content in the Gomisin G group was compared with the DMSO control group. Statistical analyses were performed using One‐way ANOVA with Bonferroni's multiple comparison test, *p < 0.05, **p < 0.01, ***p < 0.001. (c) SW1990 and PANC‐1 cells treated with Gomisin G for 24 h were analyzed by western blotting using antibodies against CDK4, CDK6, Cyclin D1, p21, p27, pRb, and Rb with β‐Actin as a loading control.

To investigate the effects of Gomisin G on cell cycle progression, pancreatic cancer cells were treated with varying concentrations of Gomisin G for 24 h and then subjected to DNA content analysis by flow cytometry (Figure 2). Gomisin G significantly increased the G1 cell content in BxPC‐3, SW1990, and PANC‐1 cells compared to the vehicle control (Figure 2a,b). The G1 content increased in a dose‐dependent manner, from approximately 41% to 54% in BxPC‐3 cells, 40% to 55% in SW1990 cells, and 44% to 59% in PANC‐1 cells (Figure 2b). Further examination of cell cycle‐related proteins revealed that Gomisin G treatment significantly reduced cyclin D1 levels while enhancing p21 and p27 in cells after 48 h of treatment. The pRb/Rb protein level was also notably reduced, whereas CDK4 and CDK6 levels remained unaffected (Figure 2c). These results indicate that Gomisin G mediates its anti‐cancer activity in pancreatic cancer cells through cell cycle arrest.

### 3.3 Gomisin G Enhances Apoptosis of Pancreatic Cancer Cells

**FIGURE 3:**
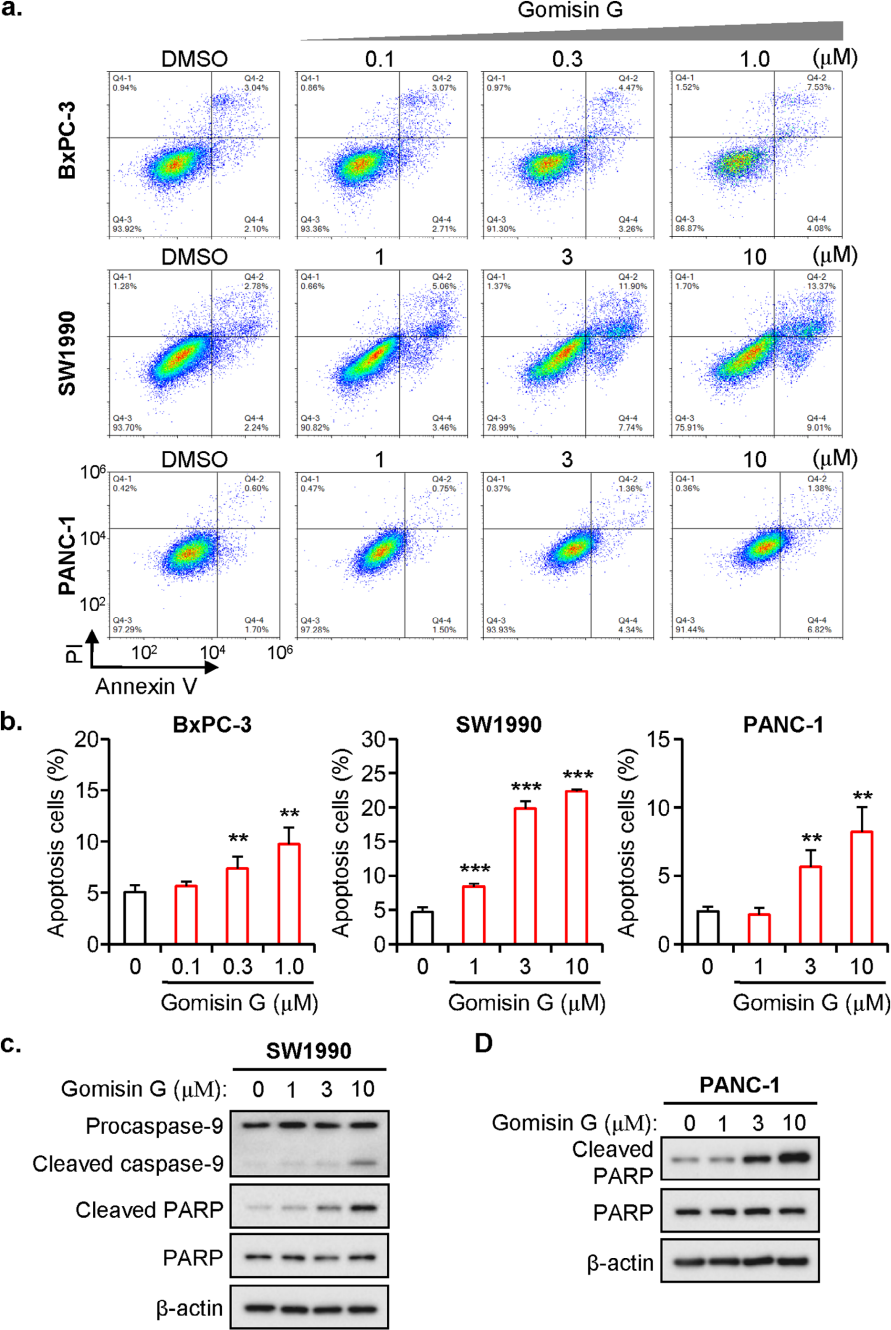
Gomisin G induces apoptosis in pancreatic cancer cells. (a) BxPC‐3, SW1990, and PANC‐1 cells were treated with Gomisin G or DMSO for 72 h, stained with annexin V and propidium iodide, and analyzed by flow cytometry using NovoExpress software. (b) Quantification of flow cytometry data from (a). Mean values from three experiments are presented. The Gomisin G group was compared with control group. Statistical analyses were performed using One‐way ANOVA with Bonferroni's multiple comparison test, *p < 0.05, ***p < 0.001, ****p < 0.0001. (c) Levels of cleaved caspase‐9, (c) leaved PARP, and PARP proteins were detected by immunoblotting in SW1990 cells treated with Gomisin G for 48 h with β‐Actin as a loading control. (d) PANC‐1 cells were treated with Gomisin G for 48 h were analyzed by SDS‐PAGE and immunoblotted with anti‐cleaved PARP and PARP antibodies, with β‐Actin as a loading control.

To determine whether Gomisin G influences cell apoptosis in pancreatic cancer cells, these cells were treated with various concentrations of Gomisin G for 72 h, stained with PI and annexin V, and analyzed by flow cytometry. Gomisin G significantly increased apoptosis in BxPC‐3, SW1990, and PANC‐1 cells compared to the vehicle control (Figure 3a,b). The induction of apoptosis was dose‐dependent, ranging from approximately 5% to 9% in BxPC‐3 cells, 8% to 22% in SW1990 cells, and 2% to 8% in PANC‐1 cells (Figure 3b). Additionally, immunoblot analysis of cleaved caspase‐9 and cleaved PARP indicated that a 10 μM dose of Gomisin G induced higher levels of apoptosis compared to DMSO in SW1990 cells (Figure 3c). Gomisin G also up‐regulated the level of cleaved PARP in PANC‐1 cells (Figure 3d). These findings suggest that Gomisin G significantly enhances apoptosis in pancreatic cancer cells.

### 3.4 Gomisin G Inhibits the Growth of PANC‐1 Tumor in Xenograft Model

**FIGURE 4:**
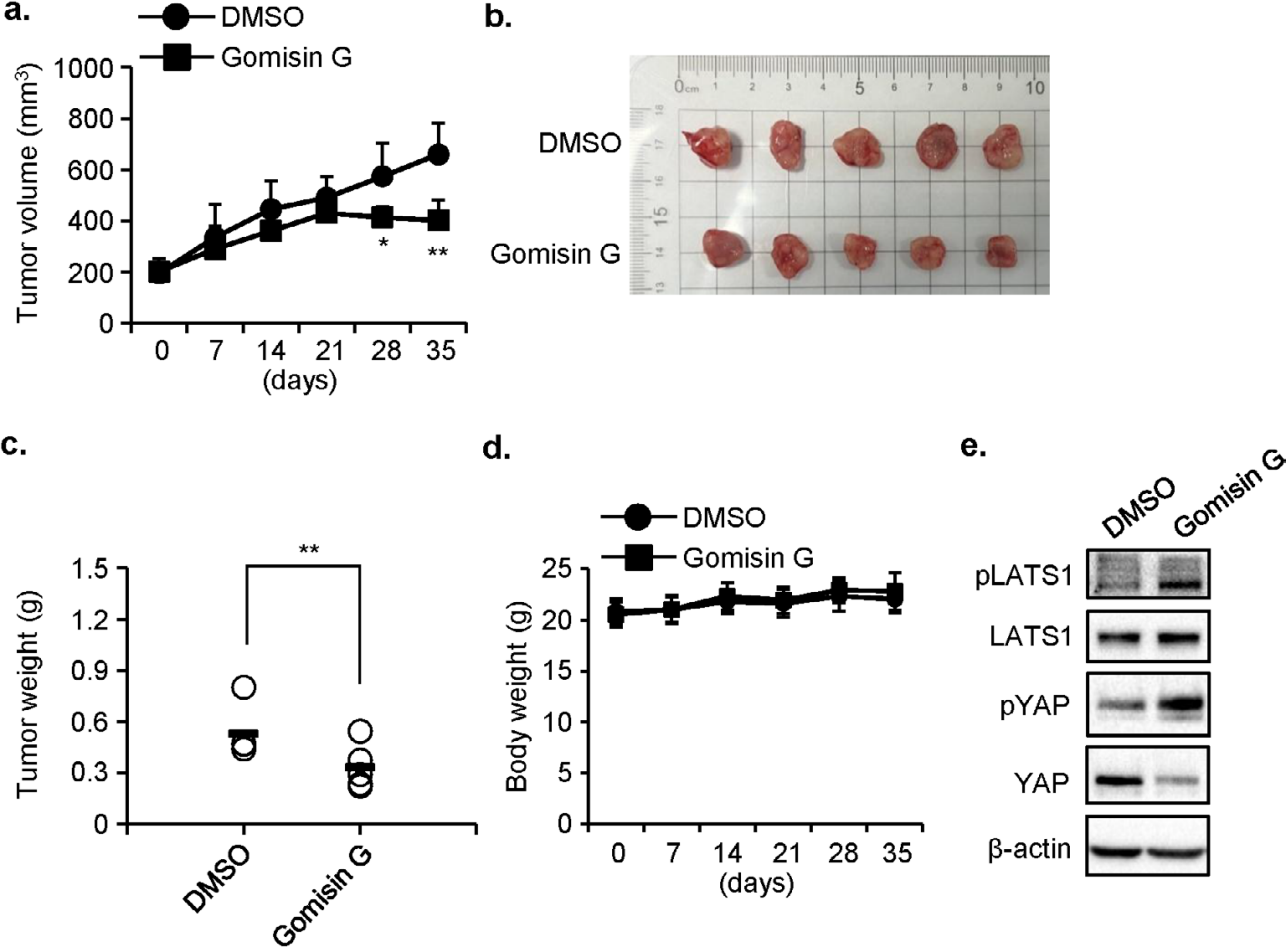
Effects of Gomisin G on pancreatic tumor growth in a xenograft mouse model. A mouse xenograft model was established by implantation of PANC‐1 cells into BALB/c nu/nu mice. When tumor volumes reached approximately 180 mm^3^, mice were orally administered either DMSO or Gomisin G (3 mg/kg). Tumor growth was monitored for 35 days (*n* = 5 each). (a) Tumor volume (calculated as width^2^ × length/2). Data are presented as mean ± standard deviation. *p < 0.05, **p < 0.01. (b) Macroscopic appearance of tumors dissected from mice euthanized at the end of the experiment. (c) Individual tumor weights. Mean values are indicated by horizontal bars. Data are presented as mean ± standard deviation. **p < 0.01. (d) Individual body weights for each treatment group. (e) Protein samples from PANC‐1 tumor tissues were analyzed by SDS‐PAGE and immunoblotted with antibodies against pLATS1, LATS1, pYAP, and YAP, withβ‐Actin as a loading control.

To test whether Gomisin G can inhibit the growth of PANC‐1 xenograft tumors, BALB/c nu/nu mice were subcutaneously injected with PANC‐1 cells. When the tumors reached a size of approximately 180 mm^3^, the mice were randomized into two groups and treated with DMSO or Gomisin G. Compared to DMSO treatment, Gomisin G significantly inhibited the growth of PANC‐1 xenograft tumors (Figure 4a–c). Moreover, the body weights of the mice were not affected by Gomisin G treatment, indicating that Gomisin G had no notable side effects (Figure 4d). Western blot analyses showed that Gomisin G treatment markedly increased the phosphorylation of both LATS1 and YAP in PANC‐1 tumor tissues, while simultaneously decreasing total YAP protein levels (Figure 4e). Collectively, these experiments indicate that Gomisin G has therapeutic effects on pancreatic tumor in a mouse xenograft model.

### 3.5 Gomisin G Enhances LATS1 Mediated Phosphorylation of YAP in Pancreatic Cancer Cells

**FIGURE 5:**
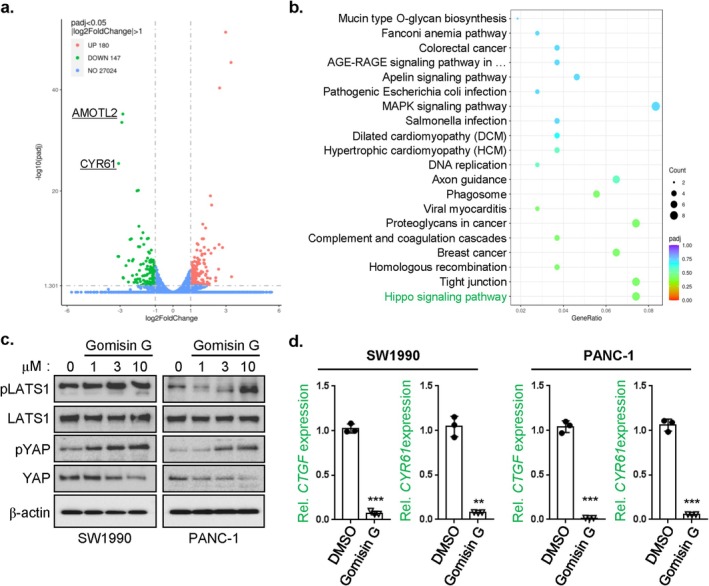
Gomisin G enhances LATS1‐mediated YAP phosphorylation in pancreatic cancer cells. (a) A volcano plot showing genes in SW1990 cells with significantly increased (red dots) or decreased (green dots) expression following Gomisin G treatment compared to the DMSO control. (b) KEGG pathway enrichment analysis indicates that differentially expressed genes are primarily enriched in the Hippo signaling pathway, tight junction, proteoglycans in cancer, and the MAPK signaling pathway. (c) SW1990 and PANC‐1 cells treated with Gomisin G for 48 h were analyzed by western blotting for pLATS1, LATS1, pYAP, and YAP, with β‐Actin as a loading control. (d) Total RNA was isolated from SW1990 and PANC‐1 cells, and relative mRNA levels of CTGF and CYR61 were analyzed by qRT‐PCR. Statistical analyses were performed using one‐way ANOVA with Bonferronis multiple comparison test, **p < 0.01, ***p < 0.001.

To determine which signaling pathways are influenced by Gomisin G in inhibiting the growth of pancreatic cancer cells, we treated the pancreatic cancer cell line SW1990 with Gomisin G and performed transcriptome sequencing. The analysis revealed that 108 genes were upregulated and 147 genes were downregulated (Figure 5a). KEGG pathway enrichment analysis indicated that these differentially expressed genes were primarily enriched in the Hippo pathway, tight junctions, proteoglycans in cancer, and the MAPK signaling pathway (Figure 5b). Notably, Gomisin G downregulated the mRNA levels of various Hippo pathway‐related target genes, including AMOTL2, CYR61, AJUBA, CCND1, ACTG1, LATS2, and CTGF.

To explore whether Gomisin G exerts anti‐cancer activity through regulation of the Hippo signaling pathway, we measured the mRNA levels of CTGF and CYR61 in SW1990 and PANC‐1 cells treated with Gomisin G using quantitative real‐time PCR analysis. The results showed that Gomisin G significantly decreased the mRNA levels of these target genes related to the Hippo pathway (Figure 5d). Western blot analysis was conducted to assess the phosphorylation and expression levels of LATS1 and YAP in these cells. The data indicated that after treatment with Gomisin G, the phosphorylation levels of LATS1 and YAP in both SW1990 and PANC‐1 cells significantly increased in a dose‐dependent manner. Conversely, the protein levels of YAP in these cells decreased dose‐dependently (Figure 5c). These findings suggest that Gomisin G suppresses YAP expression by activating LATS1 in pancreatic cancer cells.

### 3.6 Gomisin G Promotes YAP Degradation and Reduces Its Nuclear Localization in Pancreatic Cancer Cells

**FIGURE 6:**
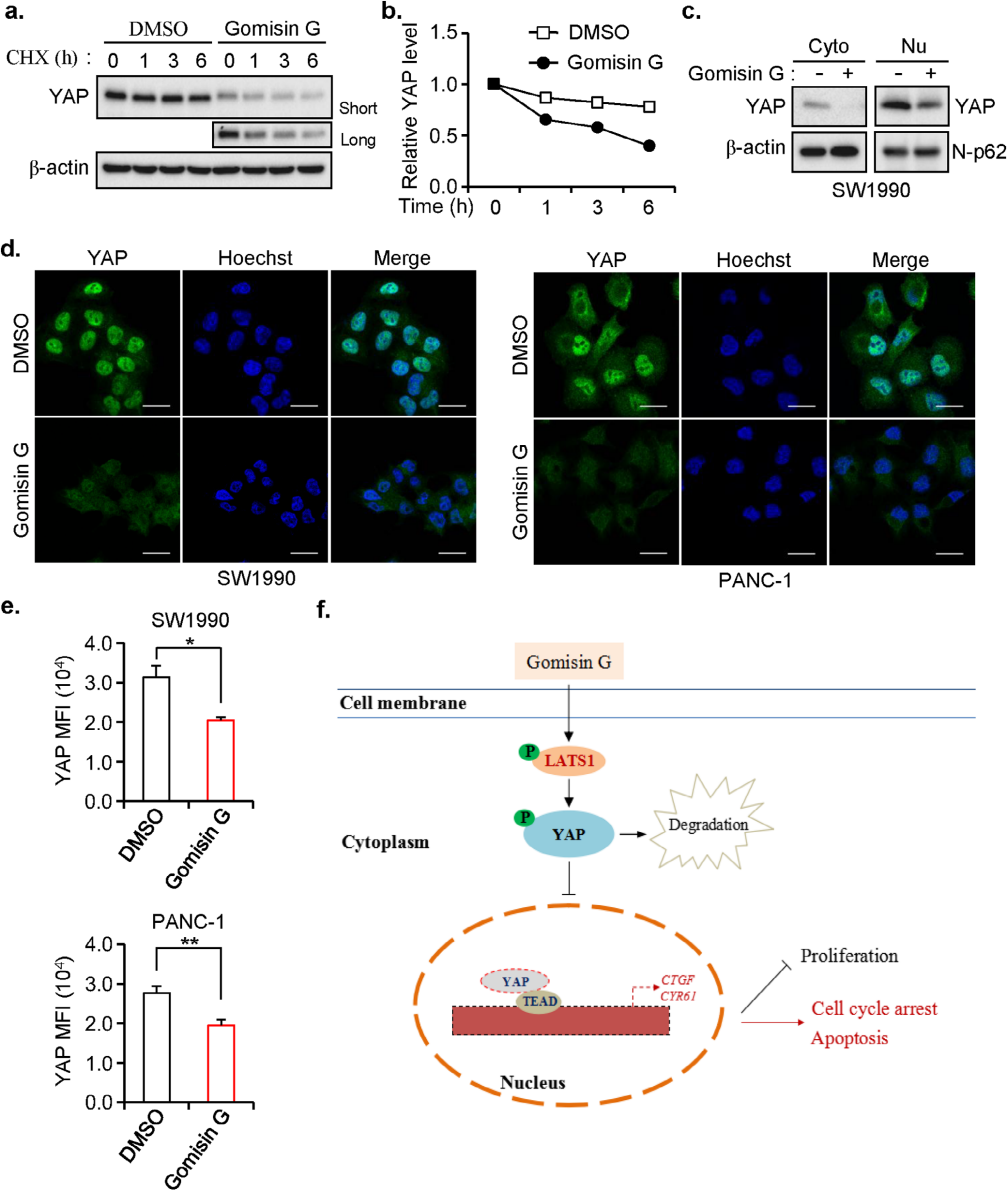
Gomisin G promotes YAP degradation and reduces nuclear localization in pancreatic cancer cells. (a) SW1990 cells treated with Gomisin G (10 μM) for 24 h were subsequently treated with cycloheximide (CHX, 10 μM) for various times, and YAP protein levels were analyzed by western blotting. (b) Relative YAP protein levels were normalized to β‐Actin and plotted over the time course of CHX treatments. (c) YAP levels in the cytoplasm and nucleus of SW1990 cells treated with Gomisin G (10 μM) for 24 h were detected by western blotting, with β‐Actin as a cytoplasmic loading control and N‐p62 as a nuclear loading control. (d) SW1990 (left half) and PANC‐1 (right half) cells were seeded on poly‐L‐lysine‐coated coverslips, treated with Gomisin G (10 μM) or DMSO for 24 h, stained with anti‐YAP antibody and Alexa Fluor 488‐conjugated secondary antibody, and observed under a confocal microscope system. Scale bars: 20 μm. (E) Quantification of mean fluorescent intensity (MFI) from (d). Mean values from three experiments are presented. The Gomisin G group was compared with the control group. Statistical analyses were performed using one‐way ANOVA with Bonferroni's multiple comparison test, *p < 0.05, **p < 0.01. (f) Proposed mechanism: Gomisin G activates LATS1 kinase, promoting YAP phosphorylation. Phosphorylated YAP is sequestered in the cytoplasm and degraded, preventing nuclear translocation and inhibiting transcriptional co‐activator activity. This results in reduced expression of downstream target genes CTGF and CYR61, leading to cell‐cycle arrest and apoptosis, ultimately suppression of tumor growth.

To investigate the impact of Gomisin G on YAP protein expression in pancreatic cancer cells, we treated SW1990 cells with Gomisin G (10 μM) for 24 h, followed by treatment with the protein synthesis inhibitor cycloheximide (CHX, 10 μM). Changes in YAP protein levels were assessed using western blot analysis. The results demonstrated that Gomisin G significantly accelerated the degradation rate of YAP in SW1990 cells compared to the DMSO control group (Figure 6a,b). Additionally, after treating SW1990 cells with Gomisin G for 24 h, we separated the proteins from the cytoplasm and nucleus. Western blot analysis showed that Gomisin G notably reduced YAP levels in both the cytoplasm and nucleus of SW1990 cells (Figure 6c). Similarly, immunofluorescence experiments indicated that Gomisin G significantly decreased the mean fluorescent intensities (MFI) of YAP and its nuclear localization in SW1990 and PANC‐1 cells (Figure 6d,e). These findings suggest that Gomisin G promotes YAP degradation and reduces its presence in the nucleus in pancreatic cancer cells.

### 3.7 Preliminary Screening of Potential Proteins Interacting With Gomisin G

To elucidate the potential protein targets of Gomisin G, we employed a multi‐platform approach utilizing four compound–target prediction tools: PharmMapper, SwissTargetPrediction, GalaxySagittarius, and the Similarity Ensemble Approach (SEA). The platforms are designed to forecast potential interactions between small molecules and proteins based on their structural and chemical characteristics. SwissTargetPrediction identified 109 potential targets, PharmMapper generated a list of 52 possible targets, GalaxySagittarius pinpointed 49 potential interactors, and SEA provided a set of 17 prospective targets. By cross‐referencing the targets predicted by these platforms and subjecting them to molecular docking validation, we narrowed down the list to 11 proteins that are likely to interact with Gomisin G. These proteins include: NQO1, PDE4D, MAPK10, ITK, PDE4B, CETP, LCK, MTOR, TNF, FKBP1A, and CCKAR (Table 1).

**TABLE 1:** Docking results of the target molecules screened by four different methods.

PDBID	Gene	S (kcal/mol)	Hydrogen bonding interactions	Arene‐H bonding interactions
1KBO	NQO1	−8.79	TrpB105	ProA68, PheB106
1MKD	PDE4D	−8.05	MetA454	IleA433, PheA469
1PMV	MAPK10	−6.33	Ser73	—
1SM2	ITK	−6.41	Asp500, Ser371, Cys442	—
1X1Z	PDE4B	−7.81	Cys432, Asn283	Phe414, Ser282
2OBD	CETP	−8.76	Cys13	—
2OF4	LCK	−7.53	Lys273	Leu251
4DR1	MTOR	−8.18	ArgA73	PheB2039
5MU8	TNF	−7.32	TyrA119	—
6YF3	FKBP1A	−7.12	Asp37, Ile56	—
7F8U	CCKAR	−9.71	Arg336, Cys196	Asn98, Phe198, Tyr176

## 4 Discussion

Pancreatic cancer is a leading cause of cancer mortality worldwide, with most patients diagnosed at advanced stages, complicating clinical treatment. Current primary treatments, including surgery and chemotherapy, have limited effectiveness in improving patient survival rates (Klein 2021). This study investigated the potential anti‐tumor activity and mechanisms of Gomisin G, a natural compound extracted from *Schisandra chinensis* fruit, on pancreatic cancer. Our findings provide valuable insights into the therapeutic potential of Gomisin G and its underlying mechanisms.

Many small molecule drugs originate from natural active compounds found in plants. Schisandra, a traditional Chinese medicine, has long been used for its pharmacological properties, including anti‐inflammatory, antiviral, and anti‐tumor effects (Chen et al. 1997; Maharjan et al. 2019; Ryu et al. 2011; Xiaoyang et al. 2015). We discovered that Gomisin G inhibited the proliferation of pancreatic cancer cell lines BxPC‐3, CFPAC‐1, SW1990, and PANC‐1 in a concentration‐dependent manner and significantly reduced the colony‐forming ability of CFPAC‐1, SW1990, and PANC‐1 cells (Figure 1). These effects are associated with cell cycle progression and apoptosis induction.

Through PI staining, Gomisin G significantly induced G1 phase arrest in BxPC‐3, SW1990, and PANC‐1 cells (Figure 2). Different stages of the cell division cycle are controlled by various cyclins, among which cyclin D1 is responsible for transitioning from G1 to S phase (Sherr 1996). Cyclin D1 itself lacks intrinsic activity to control the cell cycle and must bind to specific CDKs to exert this control (Otto and Sicinski 2017). Among these, CDK4 and CDK6 bind to cyclin D1, phosphorylating the tumor suppressor protein Rb, thus mitigating its tumor suppressive effect and allowing tumor cells to progress from G1 to S phase (Sherr et al. 2016). It is known that proteins p21 and p27, which typically inhibit CDKs, are essential regulators of cell proliferation (Guiley et al. 2019). In some cases, p27 is implicated in activating CDK4, necessitating the binding and activation of cyclin D1 by p21 or p27 in vivo (Guiley et al. 2019). Our findings indicate that Gomisin G significantly reduces the level of cyclin D1 protein in pancreatic cancer cells SW1990 and PANC‐1, and increases the levels of p21 and p27. However, Gomisin G does not affect the levels of CDK4 and CDK6 proteins (Figure 2b). Importantly, Gomisin G significantly decreases the level and activities of Rb in pancreatic cancer cells, thus halting the cancer cell cycle from G1 to S phase (Figure 2b). There are also reports that Gomisin G inhibits the growth of triple‐negative breast cancer cells by blocking AKT phosphorylation and reducing cyclin D1 (Maharjan et al. 2018).

The Hippo pathway operates as a linear kinase cascade, where complexes of MST1/2 and WW45 phosphorylate and activate LATS1/2, which in turn phosphorylates YAP and TAZ (Moroishi et al. 2015; Yu et al. 2015). YAP/TAZ, serving as primary effectors of the Hippo pathway, is considered proto‐oncoproteins closely associated with the proliferation, survival, and apoptosis of cancer cells (Moroishi et al. 2015; Zanconato et al. 2016). Activating LATS1 has been identified as a potentially effective strategy for inhibiting YAP and tumor growth. In our previous research, we conducted transcriptome sequencing analysis on SW1990 pancreatic cancer cells treated with Gomisin G and discovered that differential genes were enriched in the Hippo signaling pathway. Quantitative real‐time PCR confirmed that Gomisin G significantly reduced the mRNA levels of *CTGF* and *CYR61*, which are related to the Hippo YAP pathway (Figure 5).

We believe that Gomisin G may influence signal transduction within the Hippo pathway. Western blot and proliferation analyses reveal that Gomisin G potentiates LATS1‐mediated phosphorylation of YAP, triggering their degradation and thereby suppressing pancreatic cancer cell growth (Figure 5). Phosphorylated YAP/TAZ is targeted by proteases and ubiquitin molecules, leading to their ubiquitination and degradation (Piccolo et al. 2023; Zanconato et al. 2016). Our findings demonstrate that Gomisin G enhances the degradation rate of YAP protein expressed in SW1990 cells (Figure 6). Immunofluorescence staining further shows that Gomisin G markedly restricts the nuclear entry of YAP protein. Reportedly, C19 (a small molecule compound) can act as an activator of the tumor suppressor kinase MST1 and LATS1 to induce degradation of the Hippo transducer TAZ. Phosphorylation of MST1 and LATS1 kinase was induced by C19 in WM266 cells in a dose‐dependent manner, and reduced TAZ levels in both the cytoplasm and nucleus of the treated cells. C19 can significantly inhibit the proliferation of WM266 cells and the growth of xenograft tumor (Basu et al. 2014). However, this study did not investigate how Gomisin G affects TAZ protein function, which presents a compelling avenue for future research.

It remains unclear whether Gomisin G directly regulates LATS1 kinase activity or affects upstream target proteins to indirectly influence the Hippo YAP pathway. Initial screenings have identified 11 potential target proteins that interact with Gomisin G using a compound target prediction platform (Table 1). Notably, phosphodiesterase (PDE) family proteins PDE4B and PDE4D were identified among the potential targets. Given the potential interaction between Gomisin G and these proteins, further investigation will be undertaken.

Gomisin G, when given orally, significantly inhibited PANC‐1 xenograft growth in nude mice (Figure 4) without achieving complete tumor regression. Similar to many lignans from Schisandra, it suffers from poor aqueous solubility (Ehambarampillai and Wan 2025). As a P‐glycoprotein substrate, active efflux from enterocytes into the gut lumen curtails its intestinal uptake and systemic exposure after oral administration (Amin 2013). Its unfavorable solubility and extensive metabolic clearance call for advanced formulation technologies to harness its therapeutic capacity. Yet, the molecule's rich structural diversity, high predicted gastrointestinal absorbability, and blood–brain barrier penetrability make it a compelling prospect for downstream drug development (Ehambarampillai and Wan 2025). forthcoming research ought to focus on improving bioavailability and dissecting its broad spectrum of biological activities to fully exploit its pharmaceutical potential.

## 5 Conclusion

In summary, Gomisin G inhibits pancreatic cancer cell growth by activating LATS1, reducing YAP stability, and downregulating its transcriptional activity. Our study highlights Gomisin G as a promising candidate for pancreatic cancer treatment, warranting further investigation into its mechanisms and therapeutic potential. Future research should focus on optimizing drug delivery, exploring combination therapies, and elucidating the direct targets of Gomisin G to fully realize its clinical potential.

## Author Contributions

**Lingxiao Ye:** data curation, methodology. **Ju Huang:** data curation, methodology. **Guang Wu:** writing – review and editing, writing – original draft, supervision, project administration, funding acquisition. **Jiawei Cao:** supervision, project administration, formal analysis, funding acquisition. **Lan Li:** data curation, methodology, formal analysis, funding acquisition. **Jiayu Chen:** data curation, methodology. **Jiayi Shao:** data curation, methodology. **Shuya He:** methodology, data curation. **Licai He:** formal analysis, funding acquisition. **Haihua Gu:** writing – review and editing, supervision, project administration, funding acquisition.

## Funding

This work was supported by the grants from the Wenzhou Science and Technology Bureau of China (Y20240132 and Y2023937), grants (LZ23H160001, LQ23H160012, LTGY23H080005) from the Natural Science Foundation of Zhejiang Province, China, and was supported in part by Wenzhou Medical University Startup funds and the Key Discipline of Zhejiang Province in Medical Technology (First Class, Category A).

## Ethics Statement

The animal experiments were approved by the Institutional Animal Care and Use Committee of Wenzhou Medical University (Permit no. wydw2024‐0182).

## Consent

All authors have agreed to publish this manuscript.

## Conflicts of Interest

The authors declare no conflicts of interest.

## Data Availability

The data that support the findings of this study are available from the corresponding author upon reasonable request.
